# Structural and Optical Properties of Metal-Nitrosyl Complexes †

**DOI:** 10.3390/molecules24203638

**Published:** 2019-10-09

**Authors:** Chantal Daniel, Christophe Gourlaouen

**Affiliations:** Laboratoire de Chimie Quantique, Institut de Chimie UMR7177 CNRS-Université de Strasbourg, 4 Rue Blaise Pascal, 67070 Strasbourg, France; c.daniel@unistra.fr

**Keywords:** nitrosyl complexes, electronic structure, conformational isomerism, density functional theory, wavefunction approach

## Abstract

The electronic, structural and optical properties (including Spin–Orbit Coupling) of metal nitrosyl complexes [M(CN)_5_(NO)]^2−^ (M = Fe, Ru or Os) are investigated by means of Density Functional Theory, TD-DFT and MS-CASPT2 based on an RASSCF wavefunction. The energy profiles connecting the N-bound (η^1^-N), O-bound (η^1^-O) and side-on (η^2^-NO) conformations have been computed at DFT level for the closed shell singlet electronic state. For each structure, the lowest singlet and triplet states have been optimized in order to gain insight into the energy profiles describing the conformational isomerism in excited states. The energetics of the three complexes are similar—with the N-bound structure being the most stable—with one exception, namely the triplet ground state of the O-bound isomer for the iron complex. The conformation isomerism is highly unfavorable in the S_0_ electronic state with the occurrence of two energy barriers higher than 2 eV. The lowest bands of the spectra are assigned to MLCT_NO_/LLCT_NO_ transitions, with an increasing MLCT character going from iron to osmium. Two low-lying triplet states, T1 (MLCT_NO_/LLCT_NO_) and T2 (MLCT_NO_/IL_NO_), seem to control the lowest energy profile of the excited-state conformational isomerism.

## 1. Introduction

**Scheme 1:**
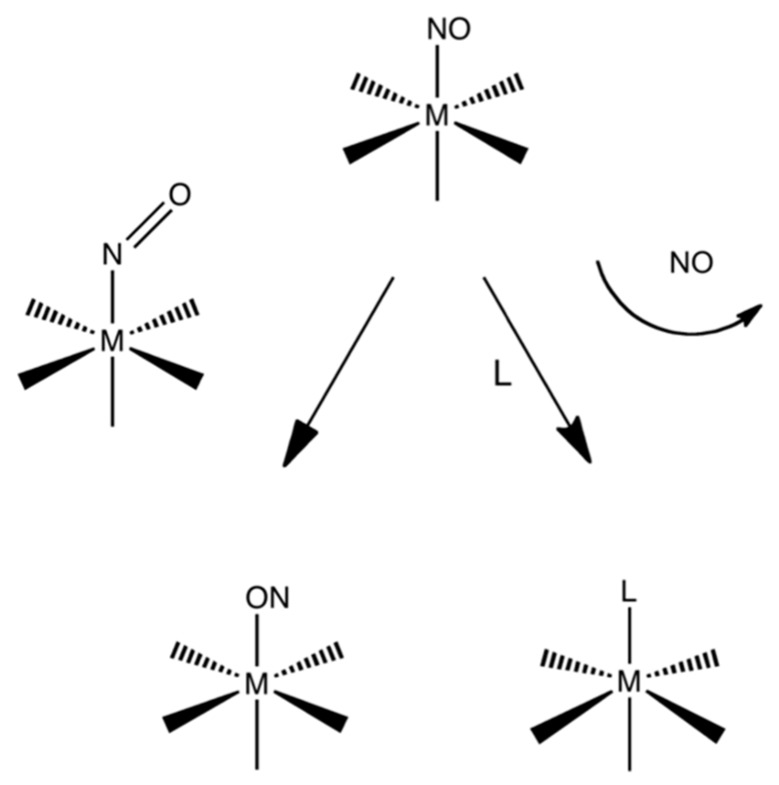
Concurrent deactivation pathways of Metal-Nitrosyl complexes (Adapted from [2] with permission of Elsevier).

Metal-Nitrosyl coordination compounds are of great interest because they exhibit two essential photo-induced primary reactions, namely NO/ON linkage isomerism and NO release (Scheme 1), which are of crucial importance in cardiovascular treatments and cancer therapies [1].

Metal-nitrosyl coordination compounds characterized by high quantum yields of NO release are particularly adapted to site-specific delivery in tumor cells within photodynamic therapy (PDT). Photo-activated nitrogen-monoxide-releasing moieties (PhotoNORMs) have been developed on the basis of Fe and Ru complexes already in the late 1990s [3] and recent reviews are dedicated to these complexes [4,5,6,7].

**Figure 1:**
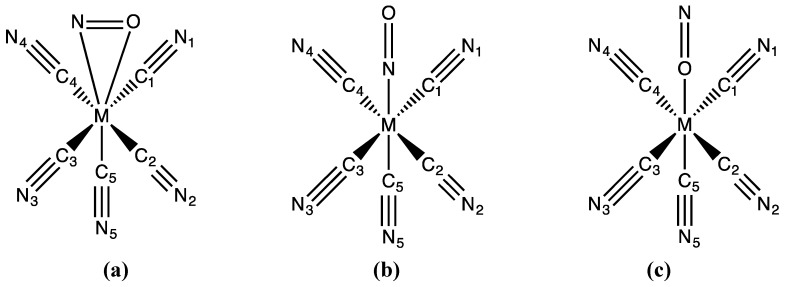
Schematic structures of the investigated [M(CN)_5_(NO)]^2−^ (M = Fe, Ru, Os) complexes in their standard N-bound (η^1^-N) (**a**) and metastable (MS) structures: flat side-on (η^2^-NO) (**b**) and reverse O-bound (η^1^-O) (**c**).

The discovery in the late 1970s of metastable isomers of sodium nitroprusside Na_2_[Fe(CN)_5_(NO)] [8] and the synthesis of related complexes, opened the way to a wealth of experimental and theoretical studies based on the development of both spectroscopic techniques and quantum chemical methods [9,10,11,12,13,14]. The co-existence of several structural isomers related to different M-NO binding modes (Figure 1) were evidenced upon irradiation of M-NO complexes [14].

Alongside their interest in the understanding of important biological processes implicating nitric oxide [3], these molecules possess the potential for efficient optical data storage based on long-lived metastable states (MS) [15,16].

Under visible irradiation, a number of {MNO}^6^ (M = Fe, Ru, Os; formally d^6^), {MNO}^8^ (M = Pt; formally d^8^) and {MNO}^10^ (M = Ni; formally d^10^) nitrosyl complexes reversibly switch between the so-called N-bound (η^1^-N) ground states (standard), O-bound (η^1^-O) (reverse) and side-on (η^2^-NO) (flat) conformations (Figure 1) [17]. For optimal efficiency, MS states should be sufficiently long-lived and the process must be thermally or photo-chemically reversible, not only at the molecular level but also in solid-state.

Most of the theoretical studies dedicated to Fe and Ru nitrosyl photo-isomerizable complexes that have recently been investigated experimentally have focused on the structural and electronic investigation of the ground state and MS isomers coupled to an analysis of the potentially photo-active states in terms of frontiers orbitals [12,18,19,20,21,22,23,24,25,26]. However, because of the non-innocent character of the nitrosyl ligand, the photo-active excited states accessible upon irradiation between 330 and 550 nm vary from pure MLCT to pure LLCT—with intermediate situations such as mixed LLCT/MC/MLCT or MC/LLCT/LC—as functions of the spectator ligands.

Light-induced linkage NO isomerism has to be initiated in the absorbing state and fast enough to compete with direct NO release or other ligands dissociation (Scheme 1). The electronic density alteration is governed by the bending of the Metal-N-O bond angle to form the intermediate MS_inter_. This can only be accomplished if the absorbing singlet state possesses a significant metal contribution (MC) that will weaken the strong M-N bond. This fragile bond will allow the isomerization and facilitate the stabilizing metal–oxygen bonding interaction in the excited state [2]. Indeed, three competitive deactivation channels of [Fe(CN)_5_(NO]^2−^ dissolved in methanol, namely Fe-NO linkage isomerism, NO release and CN dissociation, have been evidenced within the first 500 fs after 400 nm pump irradiation using transient IR spectroscopy [11]. These experiments indicate that population of the absorbing states detected at 393 nm and 520 nm at room temperature in methanol, most probably corresponding to LLCT_NO_/MLCT_NO_/MC excited states, opens the route to both ligand dissociation and Fe-NO linkage isomerism. Ultra-fast ISC to the associated low-lying triplet states cannot be excluded at this stage. However, the usual wavelength dependence of the measured quantum yields is in favor of singlet photo-reactivity. Consequently, by adjusting the initial wavelength of irradiation, it should be possible to target specific photo-active states and to control the branching ratio between the different primary reactions.

The present work aims at comparing the electronic, structural and optical properties of the [M(CN)_5_(NO)]^2−^ (M = Fe, Ru, Os) series of complexes (Figure 1). Energy profiles and critical geometries characterizing the conformational isomerism in the three molecules are investigated for the S_0_ electronic ground states, as well as for the low-lying excited states, in order to gain some insight on the mechanisms of inter-conversion.

## 2. Computational Details

Three series of calculations were undertaken. A first set was computed using ADF2013 software [27,28,29]. The structures of various isomers of the three complexes [M(CN)_5_(NO)]^2−^ (M = Fe, Ru, Os) (Figure 1) have been fully optimized for the electronic ground state ^1^A’ under the Cs symmetry constraint at the density functional theory (DFT) [30,31] level with the B3LYP functional [32] and triple-ζ basis sets [33] on all atoms. Scalar relativistic corrections were included through the zero-order relativistic approximation (ZORA) [34] and solvent effects were considered by means of the COnductor-like Screening Model COSMO [35] of methanol (ε = 32.7). Electronic ground state minima were characterized by a complete set of real frequencies. The low-lying electronic excited states were optimized at the same level of theory. The theoretical absorption spectra were computed by means of time-dependent DFT (TD-DFT) [36] performed on the ground state optimized structures. The spin–orbit coupling (SOC) has been included at a perturbative level [37]. The Tamm–Dancoff approximation (TDA) [38] was used to avoid over stabilization of the lowest triplet states.

A second set of calculations was undertaken in order to compute the Gibbs Free Energy surface leading from the standard structure to the reverse one and to locate the transition states using the GAUSSIAN 09 quantum chemistry package [39]. These calculations were carried out at the DFT level of theory with the B3LYP functional using the 6-31+G** basis set for C, N and O [40] and the Stuttgart–Dresden SDD basis sets and associated small core pseudopotentials for Fe, Ru and Os atoms [41]. Solvation (methanol) was included through a PCM model [42]. Calculations were performed without symmetry on the S_0_ electronic ground state singlet state. For comparison, calculations with the 6-311+G* basis set are provided in ESI. The results are very similar, except for one structure that we were not able to optimize using this basis set.

For the iron complex, Restricted-Active Space Self-Consistent field (RASSCF) [43], including ten electrons correlated in ten active orbitals, supplemented by multi-state complete active space perturbation theory 2nd order (MS-CASPT2) [44] calculations have been performed (on the ADF optimized structures), including solvent corrections by means of the polarized continuum model (PCM) for MeOH and using quadruple-ζ quality atomic natural relativistic correlated corrected basis sets (ANO-RCC) [45] and the Molcas 8.2 quantum chemistry package [46].

## 3. Structural and Electronic Properties

### 3.1. Structures

Some important optimized bond distances and angles of [M(CN)_5_(NO)]^2−^ (M = Fe, Ru, Os) computed with ADF in their so-called standard structure (Figure 1a) are reported in Table 1 and compared to the X-ray data for the iron [47] and ruthenium [48] complexes. Whereas the agreement between the calculated values and the experimental data is rather good for the 1st-row transition metal complex, some discrepancies are observed for 2nd-row transition metal compound, especially for the Ru-N distance, which is overestimated by 0.051 Å at this level of calculation. The structures of the 2nd- and 3rd-row complexes are very similar.

Selected optimized bond distances and angles of [M(CN)_5_(NO)]^2−^ (M = Fe, Ru, Os) in their so-called flat (Figure 1b) and reverse (Figure 1c) structures computed with ADF are reported in Table 2, together with their energy gap ΔE (in eV), with the standard structures reported in Table 1. For the iron complexes, both the lowest singlet and triplet states are reported. All the Cs-optimized structures are characterized by real frequencies in the singlet ground state. In the triplet state, symmetry is broken and the reverse structure was optimized without symmetry.

While the standard structures remain the most stable structures at this level of calculation, the reverse ones in which the nitrosyl ligand is bound to the metal center by the O atom are less stable (ΔE = 1.5–2.0 eV or 145–193 kJ Mol^−1^) for all compounds. The flat structures, where both N and O are bound to the metal atom, are at about 1.5 eV above the standard ones, as well as the reverse structure in the lowest triplet state of [Fe(CN)_5_(NO)]^2−^. This latter structure, at 1.49 eV above the standard isomer, is characterized by a bent geometry with a Fe-N-O bond angle of 128° and is nearly degenerate with the flat structure calculated at 1.46 eV.

According to the energetics reported in Table 2, the adiabatic conformational isomerism on the electronic ground state S_0_ potential energy surface (PES) seems to be easier for the ruthenium complex than for the osmium complex for which the flat and reverse structures are highly destabilized. An S_0_/T_1_ crossing characterizes the conformational isomerism pathway of the iron complex.

### 3.2. Electronic Structures and Potential Energy Profiles

One important point concerns the validity of DFT at describing correctly the electronic structure of 1st-row transition metal complexes. In particular, metal-nitrosyls may be the seat of near-degeneracies and unusual spin densities. A DFT-based theoretical study coupled to Mossbauer spectroscopy of a series of iron compounds, including nitrosyl-substituted complexes, by Ghosh A. et al. [49] has concluded that experimental isomer shifts are effectively reproduced. To further support our approach, MS-CASPT2 calculations were undertaken. At this level of theory, the S_0_ electronic state of the *standard* isomer is again the global energy minima of the three isomers of [Fe(CN)_5_(NO)]^2−^. The stability of the flat and reverse isomers is similar to that found at the DFT level, the flat being 1.62 eV and the reverse being 1.50 eV above the standard structure, the reverse structure is now slightly more stable than the flat one at the MS-CASPT2 level. This difference between DFT and MS-CASPT2 results may come from a greater multi-reference character of the reverse structure. Indeed, the standard and flat isomers are described, respectively, by one electronic configuration, weighting 81% and 85% of the total wavefunction. This weight significantly drops to 70% in the reverse case, showing a greater multi-reference character of the electronic ground state. This may be the source of the different stability of the flat and reverse isomers, with the mono-reference DFT approach being unable to evidence this contribution. The reasonable agreement between MS-CASPT2 and DFT results validates our computational scheme.

**Figure 2:**
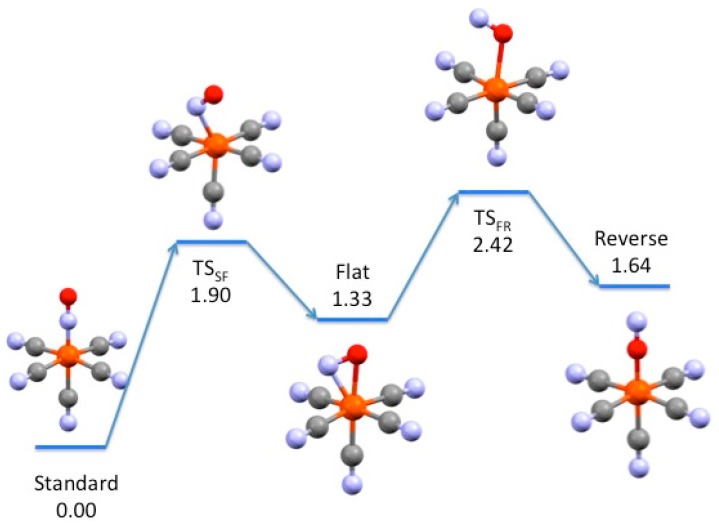
Calculated low-lying S_0_ potential energy profile of the conformational isomerism in [Fe(CN)_5_(NO)]^2−^ in methanol computed with GAUSSIAN. The potential and Gibbs free energies are reported in Table S1 of the Supplementary Information section. Energies are in eV.

The mechanism of NO isomerism in the S_0_ electronic ground state has been studied using GAUSSIAN. The potential energy profile describing the conformational isomerism for the iron complex is depicted in Figure 2. The data for the three complexes are gathered in Table 3.

The potential energy profiles are energetically similar for the three molecules, while no transition state connects the flat to reverse structures of the ruthenium complex. The transition state energies between the standard to the flat (TS_SF_) structures exceed 1.9 eV. These high energies, associated to the TS_SF_ structure, result from the breaking of the Cs symmetry and the significant lengthening of the M-N bond (from 1.66 to 2.03 Å in TS_SF_ for Fe, for instance). The overall shape of the TS_SF_ is a lacunar octahedron in which the NO ligand is close to de-coordinating from the complex. The same features characterize the structures of the obtained two transition states that connect the flat to the reverse structure (TS_FR_) with a lengthening of the M-O bond of roughly 0.5 Å ([M-O] = 2.27 Å; [M-N] = 2.94 Å). The need for a significant metal cation-NO bond weakening for ensuring the interconversions between the isomers is at the origin of the high barrier. This probably explains the difficulty in locating the TS_FR_ in the ruthenium complex. The barrier approaches the dissociation channel and all attempts to locate this transition state led either to dissociation of NO or to the rotation of the ligand around the M-O axis.

We tentatively computed the NO isomerization pathway in the lowest triplet state of the Fe complex. As mentioned above, in this state, the Cs symmetry is broken and the Fe-N-O angle is no longer linear, with a value of 138.8° for the Fe-N-O angle. The minimum standard structure in the triplet state is very similar to that of the TS_SF_ in the singlet state (0.42 eV above the singlet state). A minimum was also found for the binding through the O atom (at 0.87 eV, Table 3)—like in the reverse isomer but again in a bent structure with a Fe-O-N angle of 130.0°. Our attempts at finding a triplet state equivalent to the singlet state flat isomer converge either to the standard or reverse structure with no evidence of a transition state connecting these two structures. A scan performed on the triplet potential energy surface (PES) following the bending of the Fe-N-O angle starting from triplet standard structure did not lead to the triplet reverse isomer. At some point, a discontinuity appears on the PES and the structure evolved, resulting in the de-coordination of the NO. Starting from this structure, geometry optimization leads to a degenerate structure, with the triplet O-bound isomer at 0.87 eV above the standard one (this structure still presents imaginary frequencies, despite fulfilling the convergence criteria, which we were unable to get rid of). Searching transition states on the triplet potential energy surface requires multiconfigurational approaches in order to locate the multiple surface crossings. This is beyond the scope of the present study.

## 4. Optical Properties

### 4.1. Absorption Spectra of the Standard Structures

**Figure 3:**
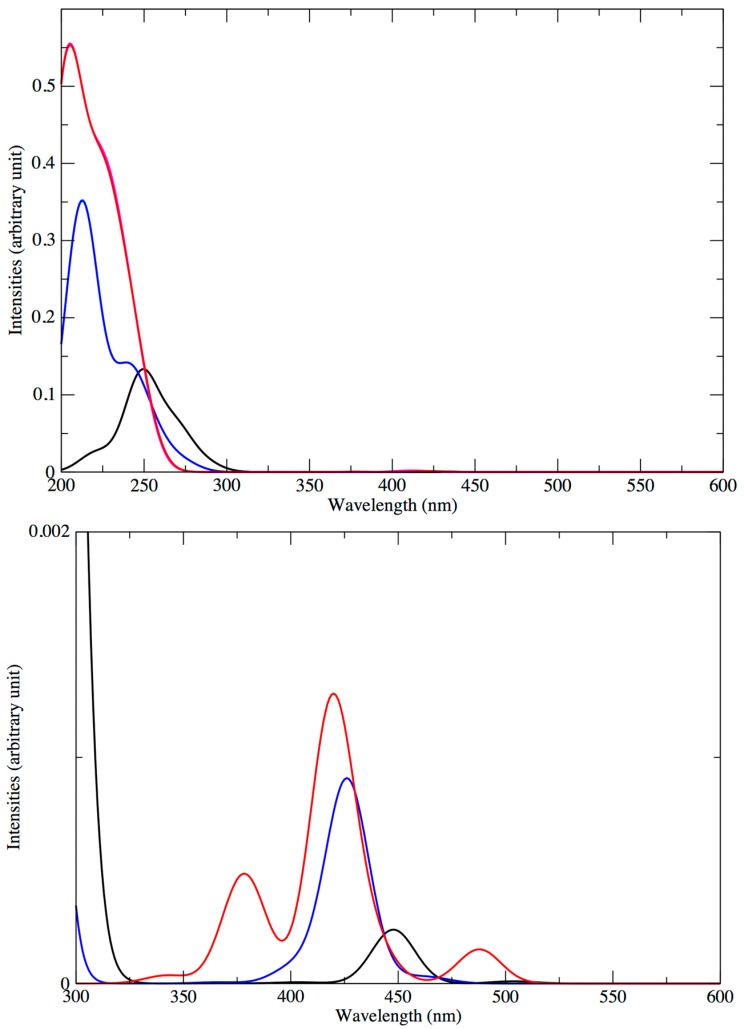
TD-DFT absorption spectra including spin–orbit coupling (SOC) of [Fe(CN)_5_(NO)]^2−^ (black), [Ru(CN)_5_(NO)]^2−^ (blue) and [Os(CN)_5_(NO)]^2−^ (red). Bottom: a zoom in the visible domain. Each transition is represented by a Gaussian function centered on the value of the transition with a width of 10 nm.

The TD-DFT absorption spectra of [Fe(CN)_5_(NO)]^2−^, [Ru(CN)_5_(NO)]^2−^ and [Os(CN)_5_(NO)]^2−^ are represented in Figure 3. The three standard complexes of C_4v_ symmetry absorb between 200 and 500 nm, with a very weak absorption starting at 500 nm. Whereas the Spin-Orbit Coupling (SOC) effects are negligible for the Fe and Ru complexes, SO splitting of the lowest band in three peaks (490, 420 and 375 nm) is observed for the Os compound. The lowest bands of the Fe and Ru complexes are calculated at 450 and 425 nm, respectively.

**Figure 4:**
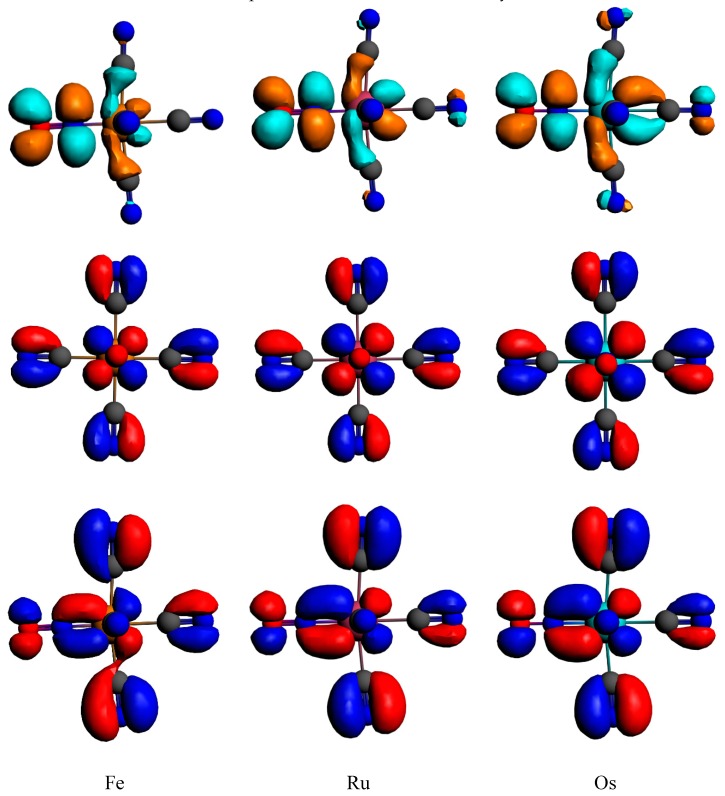
Frontier Kohn–Sham orbitals of the [M(CN)_5_(NO)]^2−^ complexes; HOMO-1 (bottom, side view), HOMO (middle, front view) and LUMO (bottom, side view).

The lowest absorbing bands in the visible domain are assigned to MLCT_NO_/LLCT_NO_ transitions described by HOMO–LUMO excitations (Figure 4), namely dπ_CN_ → π*_NO_. The MLCT character of these mixed MLCT/LLCT transition increases from Fe to Os, correlating with expanding SOC effects in the 3rd-row transition metal complex, in addition to the usual heavy atom influence.

The visible band (350–500 nm) of [Os(CN)_5_(NO)]^2−^ is shifted to the red with respect to the one of the 1st-row and 2nd-row transition metal complexes and broadened due to SOC effects. The three complexes present upper absorption peaks between 300 to 200 nm of increasing intensity and slightly blue-shifted from Fe to Os. These intense peaks are attributed to charge transfer from the cyanide π orbitals towards the LUMO. Below 200 nm, the most intense computed peak is a mixture of π_CN_ → π*_MNO_ and of π_MCN_ → π*_MNO_.

### 4.2. Low-Lying Singlet and Triplet States

Under a C_s_ symmetry constraint, the lowest singlet S_1_ is doubly degenerate with one component in the A’ symmetry point group and the other in the A” symmetry point group, corresponding to pure transitions from the HOMO to the doubly degenerate LUMO (Figure 4). The situation is trickier for the triplet states. In competition with the doubly degenerate triplet counterparts T_1_ (MLCT_NO_/LLCT_NO_) of the previous singlet states, another triplet T_2_ (MLCT_NO_/IL_NO_) of A’ symmetry is present. This T_2_ is the most stable in the iron complex and is similar to the upper-T_1_ in the ruthenium and osmium complexes. This T_2_ corresponds to a transition from the HOMO-1 to the LUMO (Figure 4). The calculated vertical transition energies to these low-lying excited states at Franck–Condon of the standard structure (Figure 1a) are reported in Table 4.

Whereas the T_1_ state calculated at 2.45 eV (Fe), 2.70 eV (Ru) and 2.82 eV (Os) is the lowest state of the Ru and Os complexes, it is above the T_2_ excited state calculated at 2.31 eV in the Fe complex. The MS-CASPT2 results are again in good agreement with the TD-DFT ones validating our approach.

**Figure 5:**
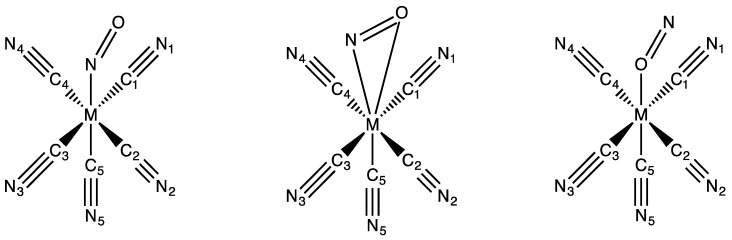
Structures of the [M(CN)_5_(NO)]^2−^ (M = Fe, Ru, Os) complexes in their low-lying electronic excited states. The NO-bent structure is on the left, the MNO-bent one is in the middle, and the ON-bent one on the right.

In order to investigate more closely the potential energy profiles of the conformational isomerism in the excited states, the structures of the five excited states reported in Table 3 have been fully optimized under Cs symmetry constraint and starting from the standard, flat and reverse geometries. In addition to the structures depicted in Figure 1, three structures represented in Figure 5 are evidenced, namely the NO-bent, MNO-bent, and ON-bent conformations.

The energetics (in eV) obtained for the different optimized structures are reported in Table 5, Table 6 and Table 7 for the Fe, Ru and Os complex, respectively. The reference energy (0.0) is given by the standard structure in its S_0_ electronic ground state.

Whereas the S_1_(A’) and T_1_(A’) excited states converge to the standard structure, the S_1_(A”) and T_1_(A”) states are more stable in the NO-bent structures and should play an important role in the conformational isomerism leading to the reverse structure. At some critical geometries, the T_2_(A’) excited state is more stable than the S_0_ electronic ground state, leading to several energy surface crossings that are potentially active in the isomerism mechanism. The T_1_(A’) excited state in the flat structure leads to de-coordination of the NO ligand.

The excited states energetics of [Ru(CN)_5_(NO)]^2−^ differ significantly to the one of the Fe complex. The de-coordination of the NO should occur via the S_1_ (A”) excited state in its flat structure. This pathway is not energetically favorable. All the excited states remain above the S_0_ electronic ground state by more than 1.5 eV for all of the structures. There is no evidence at this level of calculation of energetically favorable ON-bent structures or S_0_/T_2_(A’) potential crossings. Consequently, the mechanism of conformational isomerism seems to be much simpler for the 2nd-row complex than for the iron compound for which low-lying triplet excited states play an important role both for NO dissociation (T_1_(A’)) and in a non-adiabatic mechanism (T_2_(A’)).

Similarly to the ruthenium complex, the osmium complex does not exhibit low-lying excited state critical geometries, with the S_0_ electronic ground state remaining the lowest state for all the structures. Likewise, the S_1_(A”) excited state in the reverse conformation is dissociative for the removal of NO. When optimizing the T_2_(A’) state in the standard structure, it converges to the T_1_(A’) state, indicating a potential degeneracy of these two excited states in the osmium compound. Again the mechanism of conformational isomerism in [Os(CN)_5_(NO)]^2−^, analogously to the one proposed for [Ru(CN)_5_(NO)]^2−^, should follow an adiabatic process along the S_0_ electronic ground state potential energy surface.

## 5. Conclusions

On the basis of density functional theory (TD)-DFT and MS-CASPT2 based on an RASSCF wavefunction, we have investigated the electronic, structural and optical properties of the Group 8 metal nitrosyl complexes. The energy profiles connecting the N-bound (η^1^-N), O-bound (η^1^-O) and side-on (η^2^-NO) conformations show that conformational isomerism is unlikely in the S_0_ electronic ground state. The presence of high-energy barriers for NO interconversion (> 2 eV) is due to the fact that the NO ligand almost decoordinates to ensure the isomerization. We have shown that the energetics of the three complexes are similar, with the N-bound structure being the most stable; the main difference is the triplet ground state of the O-bound isomer for the iron complex. The optical spectra and the low-lying triplet excited states exhibit an important MLCT, LLCT and IL mixed character. The photo-induced isomerism is certainly controlled by the presence of two competing triplet states, T_1_ and T_2_. Further studies should include computation of the low-lying triplet potential energy profiles at the multi-reference level, beyond the capabilities of DFT, as well as dynamics simulation. Whereas the conformational isomerism is driven by an adiabatic process along the S_0_ potential energy surface for the Ru and Os complexes, the mechanism is clearly non-adiabatic in the case of the Fe compound involving intersystem crossings and low-lying triplet states.

## Figures and Tables

**Table 1:** Important bond distances (in Å) and bond angles (in °) of [M(CN)_5_(NO)]^2−^ (M = Fe, Ru, Os) in their so-called *standard* structure (Figure 1a) computed with ADF. (Atoms numbers according to Figure 1).

Bonds Angles	Fe	Fe (RX) ^a^	Ru	Ru (RX) ^b^	Os
M-N	1.66	1.65	1.81	1.75	1.79
M-C_1_	1.96	1.93	2.09	2.07	2.08
M-C_2_	1.96	1.93	2.09	2.07	2.08
M-C_3_	1.96	1.93	2.09	2.07	2.08
M-C_4_	1.96	1.94	2.09	2.07	2.08
M-C_5_	1.95	1.93	2.08	2.07	2.10
NO	1.13	1.13	1.14	1.15	1.14
M-N-O	179.8	178.2	179.9	177.0	179.9
N-M-C_1_	94.1	95.9	94.1	94.3	94.2
N-M-C_2_	94.0	95.5	94.1	93.3	94.1
N-M-C_3_	93.9	94.1	94.1	95.9	94.0
N-M-C_4_	94.0	96.1	94.1	95.3	94.1

^a^ Ref [47] ^b^ Ref [48].

**Table 2:** Important bond distances (in Å) and bond angles (in °) of [M(CN)_5_(NO)]^2−^ (M = Fe, Ru, Os) in their so-called flat structure (Figure 1b) and reverse structure (Figure 1c) and associated energy gap ΔE (in eV) with the standard structures. (Atoms numbers according to Figure 1).

Bonds Angles	Fe			Ru		Os	
	Flat S_0_	Reverse S_0_	Reverse ^a^ T_1_	Flat S_0_	Reverse S_0_	Flat S_0_	Reverse S_0_
M-N	1.92			2.04		2.01	
M-O	2.04	1.77	2.28	2.21	1.95	2.20	1.92
M-C_1_	1.96	1.96	1.96	2.09	2.08	2.09	2.08
M-C_2_	1.97	1.96	1.96	2.09	2.08	2.08	2.08
M-C_3_	1.95	1.96	1.95	119.3	2.08	2.08	2.08
M-C_4_	1.97	1.96	1.96	2.09	2.08	2.08	2.08
M-C_5_	1.91	1.91	1.89	2.01	2.02	2.02	2.02
NO	1.15	1.11	1.15	1.16	1.12	1.18	1.13
M-N-O	79.3			82.8		82.9	
M-O-N	67.0	179.8	128.2	65.9	179.8	64.9	179.9
N-M-C_1_	79.7	92.6	87.3	78.0	92.6	77.8	92.0
N-M-C_2_	91.0	92.6	87.2	91.5	92.4	90.9	92.0
N-M-C_3_	120.4	92.7	90.5	119.3	92.5	119.4	92.0
N-M-C_4_	91.0	92.6	88.4	91.5	92.6	90.9	92.1
E_DFT_	1.46	1.65	1.49	1.33	1.67	1.59	2.05

^a^ The structure was computed without symmetry.

**Table 3:** Calculated low-lying S_0_ potential energy profiles of the conformational isomerism for the three complexes in methanol and in the triplet state for iron. For the latter, the reference energy is the S_0_ standard structure. Gibbs Free energies are in eV.

Metal	Standard	TS_SF_	Flat	TS_FR_	Reverse
Fe	0.0	1.90	1.33	2.42	1.64
Fe (triplet)	0.42				0.87
Ru	0.0	2.00	1.33		
Os	0.0	2.30	1.54	3.01	2.02

**Table 4:** Calculated TD-DFT vertical transition energies to the low-lying excited states of [M(CN)_5_(NO)]^2−^ (M = Fe, Ru, Os) at the standard structure and MS-CASPT2 results for iron (a).

States	Fe	Fe ^a^	Ru	Os
T_1_A’	2.45	2.41	2.70	2.82
T_1_A”	2.45	2.33	2.70	2.82
T_2_A’	2.31		2.76	2.99
S_1_A’	2.79		2.92	3.03
S_1_A”	2.79		2.92	3.03

**Table 5:** Potential energy (in eV) of [Fe(CN)_5_(NO)]^2−^ calculated for the different optimized structures (Figure 1 and Figure 5) with respect to the S_0_ electronic ground state energy of the standard geometry (Figure 1a).

Structure	Standard	Flat	Reverse	NO-bent	MNO-bent	ON-bent
S_0_	0.0	1.46	1.65		2.09 ^a^	2.20 ^b^
S_1_ (A’)	2.42	3.41	2.99			
T_1_ (A’)	2.12	-NO	2.62			
T_2_ (A’)	1.77				1.86 ^a^	2.10 ^b^
S_1_ (A”)	-		2.85	2.22	2.65	
T_1_ (A”)	-			1.75	2.25	2.45

^a^ On the S_1_(A”) optimized structure; ^b^ On the T_1_(A”) optimized structure.

**Table 6:** Potential Energy (in eV) of [Ru(CN)_5_(NO)]^2−^ calculated for the different optimized structures (Figure 1 and Figure 5) with respect to the S_0_ electronic ground state energy of the standard geometry (Figure 1a).

Structure	Standard	Flat	Reverse	NO-bent	MNO-bent
S_0_	0.0	1.33	1.67		
S_1_ (A’)	2.54	3.22	3.10		
T_1_ (A’)	-		-		
T_2_ (A’)	2.19		2.58		
S_1_ (A”)	-		-NO	2.37	
T_1_ (A”)	-		2.83	2.13	2.66

**Table 7:** Potential energy (in eV) of [Os(CN)_5_(NO)]^2−^ calculated for the different optimized structures (Figure 1 and Figure 5) with respect to the S_0_ electronic ground state energy of the standard geometry (Figure 1a).

Structure	Standard	Flat	Reverse	NO-bent	MNO-bent	ON-bent
S_0_	0.0	1.59	2.05			
S_1_ (A’)	2.69	3.41	3.56			
T_1_ (A’)	2.50	3.08	-			
T_2_ (A’)	-	-	3.03			
S_1_ (A”)	-		-NO	2.54	3.31	
T_1_ (A”)	-		-	2.33	3.09	3.31

## Supplementary Materials

The following are available online.
