# Supplementary material for: Structural and Optical Properties of Metal-Nitrosyl Complexes
Source: Molecules. 2019 Oct 9;24(20):3638. doi: 10.3390/molecules24203638 (PMC6832229; doi:10.3390/molecules24203638)
Supplement: Supplementary file 1 [file molecules-24-03638-s001.pdf]

Structural and Optical Properties of Metal-Nitrosyl Complexes

Chantal Daniel and Christophe Gourlaouen*

Laboratoire de Chimie Quantique

Institut de Chimie UMR7177 CNRS-Université de Strasbourg

4 Rue Blaise Pascal 67070 Strasbourg, France

c.daniel@unistra.fr, gourlaouen@unistra.fr

Supporting Information

Table of Contents

1. Table S1………………………………………………………………….. 2
2. ADF optimized structures ……………………………………………2-14
3. GAUSSIAN optimized structures …………………………………...14-17

		Standard	TS_{SF}	*Flat*	TS_{FR}	*Reverse*
Fe	6-31+G*	0.0	1.90	1.33	2.42	1.64
	6-311+G*	0.0	1.89	1.37	2.39	1.61
Fe (triplet)	6-31+G*	0.42				0.87
	6-311+G*	0.50				0.88
Ru	6-31+G*	0.0	2.00	1.33		
	6-311+G*	0.0	1.99	1.39		
Os	6-31+G*	0.0	2.30	1.54	3.01	2.02
	6-311+G*	0.0	2.29	1.60		

Table S1. Calculated low-lying S_0 potential energy profiles of the conformational isomerism for the three complexes in methanol and in the triplet state for iron. For the latter, the reference energy is the S_0 standard structure. Gibbs Free energies are in eV. The calculation were performed with the 6-31+G* and the 6-311+G* basis sets.

ADF STRUCTURES

```
13
Iron Standard S0
 Fe        1.823744   -0.033566    0.000000
 C         2.040748   -1.976577    0.000000
 C         1.961256   -0.026964   -1.950232
 C         3.776295    0.045376    0.000000
 C         1.961256   -0.026964    1.950232
 C         1.879557    1.921070    0.000000
 N         2.155280   -3.130654    0.000000
 N         2.031094   -0.025761   -3.107927
 N         4.934778    0.091423    0.000000
 N         1.899778    3.080741    0.000000
 N         2.031094   -0.025761    3.107927
 N         0.168039   -0.105941    0.000000
 O        -0.955386   -0.159772    0.000000
13
Iron Flat S0
 Fe        1.836901   -0.034329    0.000000
 C         2.268729   -1.942464    0.000000
 C         1.859369   -0.002067   -1.968044
 C         3.747453   -0.026177    0.000000
 C         1.859369   -0.002067    1.968044
 C         2.087133    1.900093    0.000000
 N         2.522385   -3.073814    0.000000
 N         1.887558    0.020284   -3.128324
 N         4.906750    0.001160    0.000000
```

```
 N         2.241588    3.049621    0.000000
 N         1.887558    0.020284    3.128324
 N         0.074473   -0.783414    0.000000
 O        -0.172377    0.343088    0.000000
13
Iron Reverse S0
 Fe        1.873883   -0.032250    0.000000
 C         2.044330   -1.983350    0.000000
 C         1.964188   -0.028122   -1.956430
 C         3.785870    0.046303    0.000000
 C         1.964188   -0.028122    1.956430
 C         1.882307    1.926229    0.000000
 N         2.138792   -3.140757    0.000000
 N         2.012506   -0.026862   -3.116681
 N         4.945668    0.093757    0.000000
 N         1.881371    3.087539    0.000000
 N         2.012506   -0.026862    3.116681
 N        -1.008953   -0.158269    0.000000
 O         0.103714   -0.107718    0.000000
13
Iron Reverse T1
 Fe        2.058481   -0.025423    0.000000
 C         2.101128   -1.987461    0.000000
 C         2.026569   -0.027281   -1.963161
 C         3.934819    0.045762    0.000000
 C         2.026569   -0.027281    1.963161
 C         1.939361    1.933780    0.000000
 N         2.126938   -3.147251    0.000000
 N         2.008792   -0.028048   -3.123118
 N         5.094963    0.092356    0.000000
 N         1.868493    3.091688    0.000000
 N         2.008792   -0.028048    3.123118
 N        -1.373672   -0.159113    0.000000
 O        -0.220861   -0.112165    0.000000
13
Ruthenium Standard S0
 Ru        1.810703   -0.034375    0.000000
 C         2.049786   -2.107890    0.000000
 C         1.961454   -0.029075   -2.081688
 C         3.891822    0.053984    0.000000
 C         1.961454   -0.029075    2.081688
 C         1.870362    2.052125    0.000000
 N         2.193369   -3.260231    0.000000
 N         2.057262   -0.028633   -3.239012
 N         5.050506    0.103229    0.000000
 N         1.912501    3.212642    0.000000
 N         2.057262   -0.028633    3.239012
 N         0.009836   -0.112797    0.000000
 O        -1.118784   -0.164622    0.000000
13
Ruthenium Flat S0
 Ru        1.850483   -0.058093    0.000000
 C         2.282680   -2.105127    0.000000
 C         1.886136   -0.005334   -2.087133
 C         3.864373   -0.072746    0.000000
 C         1.886136   -0.005334    2.087133
 C         2.043654    2.016168    0.000000
 N         2.546166   -3.234576    0.000000
 N         1.933124    0.045509   -3.247744
 N         5.024297   -0.047916    0.000000
```

```
 N         2.189827    3.167395    0.000000
 N         1.933124    0.045509    3.247744
 N        -0.010291   -0.884327    0.000000
 O        -0.343761    0.224969    0.000000
13
Ruthenium Reverse S0
 Ru        1.889084   -0.031934    0.000000
 C         2.063234   -2.107822    0.000000
 C         1.979682   -0.028329   -2.081396
 C         3.902480    0.045565    0.000000
 C         1.979682   -0.028329    2.081396
 C         1.894643    2.052368    0.000000
 N         2.171769   -3.265873    0.000000
 N         2.041371   -0.026931   -3.242885
 N         5.063576    0.090919    0.000000
 N         1.904387    3.215499    0.000000
 N         2.041371   -0.026931    3.242885
 N        -1.168763   -0.154142    0.000000
 O        -0.054983   -0.107407    0.000000
13
Osmium Standard S0
  Os        0.103826    0.064179    0.000000
  C         1.143068   -1.739978    0.000000
  C        -0.018231   -0.023423    2.076332
  C        -1.633087   -1.107736    0.000000
  C        -0.018231   -0.023423   -2.076332
  C        -1.182797    1.701410    0.000000
  N         1.729465   -2.740855    0.000000
  N        -0.076537   -0.079173    3.233527
  N        -2.593916   -1.754051    0.000000
  N        -1.893987    2.617847    0.000000
  N        -0.076537   -0.079173   -3.233527
  N         1.586377    1.068427    0.000000
  O         2.530662    1.710115    0.000000
13
Osmium Flat S0
 Os        0.065668    0.034858   -0.008958
 C         1.221378   -1.691126   -0.074181
 C        -0.214533   -0.150480    2.055868
 C        -1.600553   -1.099359    0.114057
 C        -0.168317   -0.125279   -2.070908
 C        -1.113195    1.745021   -0.072535
 N         1.865332   -2.655614   -0.132389
 N        -0.374100   -0.252527    3.199109
 N        -2.558080   -1.751255    0.155891
 N        -1.767384    2.703107   -0.120732
 N        -0.323843   -0.227056   -3.215219
 N         1.611686    1.083560    0.723053
 O         1.851643    1.246322   -0.421940
13
Osmium Reverse S0
 Os        0.030630    0.012717    0.000000
 C         1.130703   -1.747578    0.000000
 C        -0.029781   -0.030387    2.074366
 C        -1.640435   -1.115619    0.000000
 C        -0.029781   -0.030387   -2.074366
 C        -1.192313    1.689853    0.000000
 N         1.753268   -2.728747    0.000000
 N        -0.060788   -0.056762    3.235643
 N        -2.602270   -1.764805    0.000000
```

```
 N        -1.869926    2.633886    0.000000
 N        -0.060788   -0.056762   -3.235643
 N         2.552319    1.720771    0.000000
 O         1.619234    1.087988    0.000000
13
Iron Standard S1 A'
 Fe        1.861156   -0.033306    0.000000
 C         2.047929   -1.991719    0.000000
 C         1.947052   -0.029719   -1.966523
 C         3.759662    0.045996    0.000000
 C         1.947052   -0.029719    1.966523
 C         1.883802    1.933823    0.000000
 N         2.199770   -3.141477    0.000000
 N         2.031803   -0.026005   -3.123514
 N         4.916499    0.094064    0.000000
 N         1.938859    3.092254    0.000000
 N         2.031803   -0.026005    3.123514
 N         0.085654   -0.107846    0.000000
 O        -1.047277   -0.156042    0.000000
13
Iron Standard T1 A'
 Fe        1.856258   -0.033402    0.000000
 C         2.047056   -1.992661    0.000000
 C         1.948145   -0.029537   -1.968830
 C         3.758790    0.045830    0.000000
 C         1.948145   -0.029537    1.968830
 C         1.883061    1.934925    0.000000
 N         2.209672   -3.140647    0.000000
 N         2.028140   -0.025994   -3.125876
 N         4.915482    0.093738    0.000000
 N         1.949236    3.092511    0.000000
 N         2.028140   -0.025994    3.125876
 N         0.090807   -0.107812    0.000000
 O        -1.044884   -0.156687    0.000000
13
Iron Standard T2 A'
 Fe        1.846395   -0.033313    0.000000
 C         2.044517   -1.986975    0.000000
 C         1.964239   -0.028844   -1.960094
 C         3.769371    0.046511    0.000000
 C         1.964239   -0.028844    1.960094
 C         1.882138    1.930110    0.000000
 N         2.207860   -3.135458    0.000000
 N         2.081807   -0.024702   -3.114176
 N         4.926485    0.094765    0.000000
 N         1.950169    3.088141    0.000000
 N         2.081807   -0.024702    3.114176
 N         0.039905   -0.109522    0.000000
 O        -1.098150   -0.161587    0.000000
13
Iron Flat S1 A'
 Fe        1.872183   -0.025458    0.000000
 C         2.208529   -1.967690    0.000000
 C         1.901382   -0.006549   -1.970409
 C         3.742589    0.001836    0.000000
 C         1.901382   -0.006549    1.970409
 C         2.037134    1.915557    0.000000
 N         2.507179   -3.088287    0.000000
 N         1.956888    0.019499   -3.130227
 N         4.900323    0.008738    0.000000
```

```
 N         2.230271    3.059083    0.000000
 N         1.956888    0.019499    3.130227
 N        -0.082954   -0.814483    0.000000
 O        -0.227197    0.331427    0.000000
13
Iron Flat T1 A'
 Fe        2.241914    0.022622    0.000000
 C         2.239703   -1.928244    0.000000
 C         2.173201    0.024943   -1.939328
 C         4.097924   -0.013325    0.000000
 C         2.173201    0.024943    1.939328
 C         2.166269    1.961234    0.000000
 N         2.333128   -3.088572    0.000000
 N         2.159307    0.026270   -3.103650
 N         5.258912   -0.072987    0.000000
 N         2.155449    3.124909    0.000000
 N         2.159307    0.026270    3.103650
 N        -1.288130   -0.883279    0.000000
 O        -1.710259    0.126090    0.000000
13
Iron Reverse S1 A'
 Fe        1.939734   -0.030875    0.000000
 C         2.041646   -1.987848    0.000000
 C         1.976142   -0.029326   -1.961410
 C         3.791891    0.047471    0.000000
 C         1.976142   -0.029326    1.961410
 C         1.876780    1.927788    0.000000
 N         2.122576   -3.146424    0.000000
 N         2.028426   -0.027141   -3.121620
 N         4.950647    0.097168    0.000000
 N         1.859405    3.089107    0.000000
 N         2.028426   -0.027141    3.121620
 N        -1.141712   -0.154770    0.000000
 O        -0.025444   -0.111363    0.000000
13
Iron Reverse T1 A'
 Fe        1.903509   -0.030646    0.000000
 C         2.049584   -1.983750    0.000000
 C         1.970351   -0.027717   -1.957676
 C         3.789064    0.045981    0.000000
 C         1.970351   -0.027717    1.957676
 C         1.888588    1.927879    0.000000
 N         2.185573   -3.136813    0.000000
 N         2.059529   -0.024033   -3.115232
 N         4.947085    0.093091    0.000000
 N         1.930143    3.088207    0.000000
 N         2.059529   -0.024033    3.115232
 N        -1.174710   -0.168767    0.000000
 O        -0.042300   -0.113847    0.000000
13
Iron Reverse S1 A"
 Fe        1.941685   -0.029493    0.000000
 C         2.047197   -1.981477    0.000000
 C         1.967669   -0.027927   -1.954581
 C         3.814419    0.046873    0.000000
 C         1.967669   -0.027927    1.954581
 C         1.885074    1.924415    0.000000
 N         2.155889   -3.138277    0.000000
 N         2.030179   -0.024819   -3.114792
 N         4.973193    0.093348    0.000000
```

```
 N         1.897046    3.086207    0.000000
 N         2.030179   -0.024819    3.114792
 N        -1.199634   -0.166931    0.000000
 O        -0.081057   -0.114764    0.000000
13
Iron NO-bent S1 A"
 Fe        1.830317    0.049699    0.000000
 C         1.937468   -1.912501    0.000000
 C         1.939754    0.065648   -1.965194
 C         3.744856   -0.032462    0.000000
 C         1.939754    0.065648    1.965194
 C         1.958934    2.013967    0.000000
 N         2.046155   -3.067461    0.000000
 N         2.046383    0.070777   -3.120122
 N         4.898456   -0.132838    0.000000
 N         2.094689    3.166057    0.000000
 N         2.046383    0.070777    3.120122
 N         0.055510   -0.104325    0.000000
 O        -0.876149   -0.753414    0.000000
13
Iron NO-bent T1 A"
  Fe        1.817588    0.057612    0.000000
  C         1.930839   -1.907486    0.000000
  C         1.930914    0.066945   -1.970963
  C         3.759034   -0.035326    0.000000
  C         1.930914    0.066945    1.970963
  C         1.967374    2.029981    0.000000
  N         2.025843   -3.063172    0.000000
  N         2.045346    0.072009   -3.124988
  N         4.912763   -0.134190    0.000000
  N         2.121521    3.179454    0.000000
  N         2.045346    0.072009    3.124988
  N         0.052428   -0.110342    0.000000
  O        -0.844087   -0.807851    0.000000
13
Iron MNO-bent S1 A"
 Fe        1.978115    0.003691    0.000000
 C         2.217029   -1.966987    0.000000
 C         1.978722    0.035072   -1.962304
 C         3.852384   -0.078871    0.000000
 C         1.978722    0.035072    1.962304
 C         1.970796    1.947142    0.000000
 N         2.365862   -3.118199    0.000000
 N         1.993711    0.062731   -3.123546
 N         5.006846   -0.193253    0.000000
 N         2.040026    3.107065    0.000000
 N         1.993711    0.062731    3.123546
 N        -0.002287   -0.701347    0.000000
 O        -0.690756    0.189637    0.000000
13
Iron MNO-bent T1 A"
 Fe        1.907984   -0.012305    0.000000
 C         2.234178   -1.970595    0.000000
 C         1.930230    0.008553   -1.971338
 C         3.819670   -0.040284    0.000000
 C         1.930230    0.008553    1.971338
 C         2.009940    1.946694    0.000000
 N         2.444636   -3.111143    0.000000
 N         1.985422    0.040563   -3.130410
 N         4.977503   -0.090431    0.000000
```

```
 N         2.186409    3.094000    0.000000
 N         1.985422    0.040563    3.130410
 N         0.014048   -0.737405    0.000000
 O        -0.561650    0.245088    0.000000
13
Iron ON-bent T1 A"
 Fe        1.914300    0.272077    0.000000
 C         2.411661   -1.609564    0.000000
 C         1.921347    0.261994   -1.961684
 C         3.693344    0.858981    0.000000
 C         1.921347    0.261994    1.961684
 C         1.451391    2.176953    0.000000
 N         2.751076   -2.720771    0.000000
 N         1.964431    0.262124   -3.122890
 N         4.777226    1.273812    0.000000
 N         1.169545    3.304961    0.000000
 N         1.964431    0.262124    3.122890
 N        -1.139731    0.602753    0.000000
 O        -0.193916    0.014019    0.000000
13
Ruthenium Standard S1 A'
 Ru        1.854117   -0.033197    0.000000
 C         2.037629   -2.120253    0.000000
 C         1.947229   -0.029459   -2.089648
 C         3.844491    0.052654    0.000000
 C         1.947229   -0.029459    2.089648
 C         1.857661    2.062047    0.000000
 N         2.179568   -3.272391    0.000000
 N         2.070845   -0.024771   -3.244681
 N         5.004158    0.102813    0.000000
 N         1.900566    3.222106    0.000000
 N         2.070845   -0.024771    3.244681
 N        -0.072163   -0.117400    0.000000
 O        -1.208759   -0.168728    0.000000
13
Ruthenium Standard T2 A'
 Ru        1.834605   -0.034055    0.000000
 C         2.050665   -2.110097    0.000000
 C         1.964001   -0.028622   -2.083344
 C         3.860529    0.053256    0.000000
 C         1.964001   -0.028622    2.083344
 C         1.871837    2.052964    0.000000
 N         2.236845   -3.256686    0.000000
 N         2.108404   -0.023211   -3.236082
 N         5.018634    0.103255    0.000000
 N         1.958479    3.211315    0.000000
 N         2.108404   -0.023211    3.236082
 N        -0.129940   -0.119302    0.000000
 O        -1.270766   -0.171533    0.000000
13
Ruthenium Flat S1 A'
 Ru        1.876921   -0.054966    0.000000
 C         2.220385   -2.125683    0.000000
 C         1.929161   -0.012381   -2.093484
 C         3.848860   -0.026204    0.000000
 C         1.929161   -0.012381    2.093484
 C         1.987620    2.001179    0.000000
 N         2.526406   -3.245051    0.000000
 N         2.021702    0.050333   -3.250589
 N         5.007834   -0.044945    0.000000
```

```
 N         2.141595    3.151963    0.000000
 N         2.021702    0.050333    3.250589
 N        -0.189625   -0.903832    0.000000
 O        -0.400497    0.236083    0.000000
13
Ruthenium Flat T1 A'
 Ru        1.870947   -0.052120    0.000000
 C         2.224970   -2.125297    0.000000
 C         1.930962   -0.011957   -2.099747
 C         3.836101   -0.034979    0.000000
 C         1.930962   -0.011957    2.099747
 C         1.988264    2.022777    0.000000
 N         2.543814   -3.241047    0.000000
 N         2.027989    0.018374   -3.257480
 N         4.996106   -0.033338    0.000000
 N         2.166631    3.169087    0.000000
 N         2.027989    0.018374    3.257480
 N        -0.187485   -0.907018    0.000000
 O        -0.399266    0.237494    0.000000
13
Ruthenium Reverse S1 A'
 Ru        1.992062   -0.025824    0.000000
 C         2.039019   -2.105407    0.000000
 C         1.973987   -0.027184   -2.080163
 C         3.908640    0.044429    0.000000
 C         1.973987   -0.027184    2.080163
 C         1.878488    2.050226    0.000000
 N         2.035614   -3.268723    0.000000
 N         1.950461   -0.028599   -3.243561
 N         5.071904    0.088789    0.000000
 N         1.779375    3.209376    0.000000
 N         1.950461   -0.028599    3.243561
 N        -1.296248   -0.174452    0.000000
 O        -0.184707   -0.116984    0.000000
13
Ruthenium Reverse T2 A'
 Ru        1.948549   -0.032873    0.000000
 C         2.060450   -2.105404    0.000000
 C         1.975395   -0.031235   -2.077000
 C         3.909593    0.052305    0.000000
 C         1.975395   -0.031235    2.077000
 C         1.884526    2.043196    0.000000
 N         2.160515   -3.264642    0.000000
 N         2.030043   -0.027379   -3.239214
 N         5.069486    0.102764    0.000000
 N         1.887430    3.206760    0.000000
 N         2.030043   -0.027379    3.239214
 N        -1.350609   -0.135914    0.000000
 O        -0.224506   -0.116025    0.000000
13
Ruthenium Reverse S1 A"
 Ru        2.102679    0.120805    0.000000
 C         1.767737   -1.915111    0.000000
 C         2.061386    0.128349   -2.081559
 C         3.983855   -0.190208    0.000000
 C         2.061386    0.128349    2.081559
 C         1.994960    2.170184    0.000000
 N         1.298310   -2.985382    0.000000
 N         1.996591    0.133582   -3.244800
 N         5.125949   -0.432994    0.000000
```

```
 N         1.658287    3.285766    0.000000
 N         1.996591    0.133582    3.244800
 N        -0.965983   -1.430101    0.000000
 O        -0.626608   -0.384080    0.000000
13
Ruthenium Reverse T1 A"
 Ru        1.957554   -0.034929    0.000000
 C         2.065089   -2.105386    0.000000
 C         1.973424   -0.033672   -2.074445
 C         3.911394    0.055033    0.000000
 C         1.973424   -0.033672    2.074445
 C         1.882006    2.039450    0.000000
 N         2.158769   -3.265326    0.000000
 N         2.017168   -0.030618   -3.237321
 N         5.071630    0.110387    0.000000
 N         1.874252    3.203196    0.000000
 N         2.017168   -0.030618    3.237321
 N        -1.360929   -0.115447    0.000000
 O        -0.239089   -0.113718    0.000000
13
Ruthenium NO-bent S1 A"
 Ru        1.849259    0.012889    0.000000
 C         2.038155   -2.081473    0.000000
 C         1.951773    0.031698   -2.091690
 C         3.849530   -0.044626    0.000000
 C         1.951773    0.031698    2.091690
 C         1.854780    2.088816    0.000000
 N         2.249885   -3.223557    0.000000
 N         2.055772    0.039115   -3.247904
 N         5.003820   -0.166003    0.000000
 N         1.887800    3.250355    0.000000
 N         2.055772    0.039115    3.247904
 N        -0.079560   -0.269881    0.000000
 O        -0.939542   -1.016627    0.000000
13
Ruthenium NO-bent T1 A"
 Ru        1.845328    0.014633    0.000000
 C         2.032459   -2.085134    0.000000
 C         1.951925    0.030962   -2.094537
 C         3.856228   -0.051727    0.000000
 C         1.951925    0.030962    2.094537
 C         1.853696    2.096097    0.000000
 N         2.240093   -3.227779    0.000000
 N         2.062948    0.043969   -3.249915
 N         5.010378   -0.171201    0.000000
 N         1.886192    3.257152    0.000000
 N         2.062948    0.043969    3.249915
 N        -0.077569   -0.267960    0.000000
 O        -0.924732   -1.031908    0.000000
13
Ruthenium MNO-bent T1 A"
 Ru        1.970329   -0.007995    0.000000
 C         2.182275   -2.119420    0.000000
 C         2.012257    0.050045   -2.091750
 C         3.930879   -0.155386    0.000000
 C         2.012257    0.050045    2.091750
 C         1.874606    2.057763    0.000000
 N         2.322361   -3.272451    0.000000
 N         2.055721    0.092839   -3.252568
 N         5.074569   -0.362550    0.000000
```

N	1.832074	3.220335	0.000000
N	2.055721	0.092839	3.252568
N	-0.079110	-0.839771	0.000000
O	-0.833575	0.004595	0.000000

13
Osmium Standard S1 A'

Os	0.087084	0.052231	0.000000
C	1.170994	-1.732942	0.000000
C	-0.021192	-0.021572	2.074580
C	-1.579203	-1.076082	0.000000
C	-0.021192	-0.021572	-2.074580
C	-1.167231	1.721958	0.000000
N	1.760849	-2.731757	0.000000
N	-0.134250	-0.099477	3.227179
N	-2.539293	-1.725673	0.000000
N	-1.876017	2.640201	0.000000
N	-0.134250	-0.099477	-3.227179
N	1.656417	1.112050	0.000000
O	2.610579	1.753691	0.000000

13
Osmium Standard T1 A'

Os	0.088221	0.052939	0.000000
C	1.168163	-1.736213	0.000000
C	-0.017472	-0.019236	2.078019
C	-1.581248	-1.077249	0.000000
C	-0.017472	-0.019236	-2.078019
C	-1.171122	1.720590	0.000000
N	1.749205	-2.739819	0.000000
N	-0.127824	-0.095395	3.230600
N	-2.541220	-1.726375	0.000000
N	-1.887491	2.632518	0.000000
N	-0.127824	-0.095395	-3.230600
N	1.654564	1.111061	0.000000
O	2.610212	1.754156	0.000000

13
Osmium Flat S1 A'

Os	-0.087882	0.020530	0.000000
C	-0.049952	0.068685	2.080097
C	-0.044415	-2.077425	0.000000
C	1.875499	-0.287632	0.000000
C	0.330046	2.036715	0.000000
C	-0.049952	0.068685	-2.080097
N	0.044187	0.118906	3.236743
N	0.034627	-3.233990	0.000000
N	3.018210	-0.473449	0.000000
N	0.044187	0.118906	-3.236743
N	0.624450	3.158266	0.000000
N	-2.172462	-0.501574	0.000000
O	-2.196969	0.686720	0.000000

13
Osmium Flat T1 A'

Os	-0.097683	0.017920	0.000000
C	-0.036842	0.077080	2.083394
C	-0.036433	-2.077943	0.000000
C	1.866291	-0.280683	0.000000
C	0.352396	2.041691	0.000000
C	-0.036842	0.077080	-2.083394
N	0.051021	0.123121	3.240329
N	0.072284	-3.232005	0.000000
N	3.011399	-0.454082	0.000000

```
 N         0.051021    0.123121   -3.240329
 N         0.683938    3.152211    0.000000
 N        -2.172292   -0.511223    0.000000
 O        -2.206062    0.684568    0.000000
13
Osmium Reverse S1 A'
 Os       -0.033439    0.028431    0.000000
 C        -0.041183    0.033916    2.071895
 C         1.370058    1.557030    0.000000
 C        -1.462942    1.327544    0.000000
 C        -1.413062   -1.519621    0.000000
 C        -0.041183    0.033916   -2.071895
 N        -0.038716    0.029694    3.234391
 N         2.183963    2.386586    0.000000
 N        -2.322371    2.110689    0.000000
 N        -0.038716    0.029694   -3.234391
 N        -2.157855   -2.411702    0.000000
 N         2.376119   -2.106172    0.000000
 O         1.524298   -1.366153    0.000000
13
Osmium Reverse T2 A'
 Os       -0.006759    0.007369    0.000000
 C        -0.047087    0.043301    2.069087
 C         1.349022    1.571652    0.000000
 C        -1.461619    1.337779    0.000000
 C        -1.443791   -1.483074    0.000000
 C        -0.047087    0.043301   -2.069087
 N        -0.088057    0.078481    3.230022
 N         2.091136    2.466227    0.000000
 N        -2.318112    2.120748    0.000000
 N        -0.088057    0.078481   -3.230022
 N        -2.268303   -2.302239    0.000000
 N         2.366391   -2.158925    0.000000
 O         1.519933   -1.387121    0.000000
13
Osmium Reverse S1 A"
 Os       -0.198286   -0.000692    0.000000
 C        -0.162829   -0.041116    2.068168
 C         1.455388    1.184178    0.000000
 C        -1.228622    1.612324    0.000000
 C        -1.574924   -1.517480    0.000000
 C        -0.162829   -0.041116   -2.068168
 N        -0.137518   -0.079333    3.232176
 N         2.516104    1.676296    0.000000
 N        -1.807010    2.626629    0.000000
 N        -0.137518   -0.079333   -3.232176
 N        -2.198224   -2.501691    0.000000
 N         3.236326   -0.901780    0.000000
 O         2.280128   -1.454156    0.000000
13
Osmium NO-bent S1 A"
 Os        0.037459   -0.070524    0.000000
 C         0.119947   -0.009298   -2.085575
 C        -1.560765    1.273272    0.000000
 C         1.165302    1.600839    0.000000
 C         1.748030   -1.237204    0.000000
 C         0.119947   -0.009298    2.085575
 N         0.174382    0.037244   -3.243454
 N        -2.420763    2.052316    0.000000
 N         1.708930    2.624803    0.000000
```

```
 N        0.174382    0.037244    3.243454
 N        2.732259   -1.853611    0.000000
 N       -1.300131   -1.441518    0.000000
 O       -2.410873   -1.745729    0.000000
13
Osmium NO-bent T1 A"
 Os       0.035504   -0.071052    0.000000
 C        0.121802   -0.008044   -2.086891
 C       -1.565567    1.273471    0.000000
 C        1.163825    1.608004    0.000000
 C        1.747940   -1.242106    0.000000
 C        0.121802   -0.008044    2.086891
 N        0.187321    0.039785   -3.243947
 N       -2.426034    2.051306    0.000000
 N        1.712519    2.628736    0.000000
 N        0.187321    0.039785    3.243947
 N        2.730939   -1.859738    0.000000
 N       -1.295739   -1.443334    0.000000
 O       -2.409295   -1.743179    0.000000
13
Osmium MNO-bent S1 A"
 Os      -0.035540    0.042017    0.000000
 C        0.014151    0.122614    2.077673
 C       -0.091806   -2.067237    0.000000
 C        1.896119   -0.379319    0.000000
 C        0.260346    2.076664    0.000000
 C        0.014151    0.122614   -2.077673
 N        0.065692    0.170286    3.237381
 N       -0.113812   -3.226922    0.000000
 N        2.996647   -0.749972    0.000000
 N        0.065692    0.170286   -3.237381
 N        0.495383    3.215615    0.000000
 N       -2.101518   -0.510424    0.000000
 O       -2.686894    0.482575    0.000000
13
Osmium MNO-bent T1 A"
 Os      -0.044905    0.039350    0.000000
 C        0.006854    0.124574    2.079675
 C       -0.080457   -2.074040    0.000000
 C        1.901220   -0.372076    0.000000
 C        0.268386    2.074563    0.000000
 C        0.006854    0.124574   -2.079675
 N        0.072687    0.187652    3.237446
 N       -0.091009   -3.233659    0.000000
 N        3.005247   -0.729059    0.000000
 N        0.072687    0.187652   -3.237446
 N        0.525183    3.208198    0.000000
 N       -2.088665   -0.521292    0.000000
 O       -2.659005    0.486080    0.000000
13
Osmium ON-bent T1 A"
 Os      -0.115882   -0.017191    0.000000
 C       -0.113503   -0.034725    2.071284
 C        1.445790    1.339462    0.000000
 C       -1.303888    1.502655    0.000000
 C       -1.529022   -1.499702    0.000000
 C       -0.113503   -0.034725   -2.071284
 N       -0.109726   -0.050790    3.234582
 N        2.403351    2.001825    0.000000
 N       -1.957702    2.466260    0.000000
```

N	-0.109726	-0.050790	-3.234582
N	-2.279380	-2.389594	0.000000
N	2.904403	-1.206520	0.000000
O	1.789991	-1.339214	0.000000

GAUSSIAN STRUCTURES

13

Iron Standard S0

Fe	0.00149500	-0.00106700	-0.09336900
C	-0.03222200	0.02103400	1.86410100
C	-1.75416100	0.85771400	0.02002800
C	0.85659200	1.75411800	0.05268600
C	1.75258100	-0.85664900	0.09511900
C	-0.85785400	-1.75326800	0.06284700
N	-0.05222500	0.03386700	3.03384600
N	2.80100800	-1.36744800	0.19103400
N	-1.37158600	-2.80173800	0.14115000
N	1.36881800	2.80371400	0.12573200
N	-2.80414500	1.37165900	0.07265600
N	0.02861100	-0.01928200	-1.73124100
O	0.04726700	-0.03192100	-2.87166600

13

Iron TSSF S0

Fe	0.13069500	-0.07567300	0.02902000
C	0.59009900	0.36995900	-1.73711000
C	-1.18745200	-1.36371300	-0.65613100
C	1.33862400	-1.54860400	0.38180500
C	1.50056700	1.17321200	0.69437100
C	-1.12784200	1.43351600	-0.30005500
N	0.93624600	0.59019600	-2.83558500
N	2.33298100	1.90091600	1.08540500
N	-1.87312000	2.32266900	-0.47067700
N	2.06244900	-2.44258400	0.61587100
N	-1.95786000	-2.13592100	-1.08678900
N	-0.94872300	0.40034100	1.68559200
O	-1.74323300	-0.35850400	1.99893500

13

Iron Flat S0

Fe	-0.00016900	0.02806300	-0.12906000
C	0.00035300	-0.17615400	1.76755000
C	-1.38040600	1.40923600	0.04432800
C	1.37930400	1.41018200	0.04440300
C	1.48359600	-1.27200700	-0.02254300
C	-1.48287100	-1.27288100	-0.02213100
N	0.00074600	-0.27164900	2.93388700
N	2.36724000	-2.03798600	0.03986000
N	-2.36594400	-2.03946500	0.04094100
N	2.19600000	2.24440400	0.13982500
N	-2.19754400	2.24305200	0.13952600
N	0.00022500	-0.74637000	-1.85226200
O	-0.00006700	0.36702500	-2.20081400

13

Iron TSFR S0

Fe	0.10332900	0.07948200	0.00510200
C	1.01337600	0.28777500	1.63121000
C	-1.54685000	-0.07145200	1.04459000
C	-0.23786400	1.98655900	-0.24109000

```
 C                  1.64660700    0.20484000   -1.19599600
 C                  0.52600500   -1.82747400    0.22843600
 N                  1.60416000    0.46464300    2.62974700
 N                  2.55779000    0.28914400   -1.93100000
 N                  0.77385000   -2.96767100    0.35097700
 N                 -0.43139800    3.13400900   -0.39637200
 N                 -2.56153500   -0.16548200    1.62738100
 O                 -1.17058000   -0.93043800   -1.58079300
 N                 -2.18994800   -0.48385900   -1.75061900
13
Iron Reverse S0
 Fe                -0.00060500    0.00018400   -0.05436100
 C                  0.03567000   -0.02895700    1.86383700
 C                 -1.53095900    1.21983800    0.09791900
 C                  1.22394800    1.53034400    0.04912800
 C                  1.53359200   -1.22166100    0.00102400
 C                 -1.21846800   -1.53465800    0.04870100
 N                  0.05817700   -0.04746800    3.03450200
 N                  2.44981100   -1.95160100    0.02040000
 N                 -1.94702900   -2.45095200    0.09594500
 N                  1.95614400    2.44363500    0.09789000
 N                 -2.44506900    1.94860300    0.17478400
 O                 -0.03787600    0.03041200   -1.80130200
 N                 -0.06403000    0.05242500   -2.92921600
13
Ruthenium Standard S0
 Ru                -0.00178000   -0.00154900   -0.08852300
 C                  0.02753000    0.01868800    1.98834600
 C                  0.06788100    2.06842800    0.06185300
 C                  2.06890300   -0.06511900    0.05417500
 C                 -0.06451300   -2.06832500    0.10294900
 C                 -2.06774300    0.06783400    0.11032100
 N                  0.04309300    0.03013000    3.15722700
 N                 -0.09694600   -3.23308900    0.21280900
 N                 -3.23206200    0.10899500    0.22187300
 N                  3.23590900   -0.09799900    0.13661900
 N                  0.10921600    3.23474800    0.14990800
 N                 -0.02666500   -0.01843300   -1.86546600
 O                 -0.04272900   -0.02891500   -3.01270600
13
Ruthenium TSSF S0
 Ru                 0.04794900   -0.10080600    0.01079900
 C                  0.62748500    0.43589400   -1.78027100
 C                 -1.58417400    1.02904900   -0.59276000
 C                 -1.18191900   -1.72637000   -0.33544500
 C                  1.56515000   -1.44633200    0.43774700
 C                  1.46281900    1.37179800    0.45734900
 N                  0.98727700    0.68640300   -2.86811100
 N                  2.40506000   -2.23564100    0.65695100
 N                  2.26633900    2.18098900    0.72587200
 N                 -1.84860300   -2.67369500   -0.52366500
 N                 -2.51911300    1.64744500   -0.93466400
 N                 -0.51825100    0.68273800    1.88431900
 O                 -1.60686000    0.55419300    2.22752600
13
Ruthenium Flat S0
 Ru                -0.02200500   -0.00709800   -0.13098800
 C                  0.22303800    0.07046600    1.85513000
 C                  0.54321800   -2.00751200   -0.09619200
 C                 -1.95052700   -0.60904200    0.33092500
```

```
 C                 -0.69532400    1.95951000   -0.09697200
 C                  1.95587200    0.61009500    0.07643800
 N                  0.33606600    0.10627100    3.01833600
 N                 -1.09067400    3.06218300   -0.07562400
 N                  3.06659200    0.95692800    0.19498200
 N                 -3.03688500   -0.94676700    0.60539800
 N                  0.84772800   -3.13863600   -0.07427000
 N                  0.56501800    0.17572800   -2.03005000
 O                 -0.53804600   -0.16734400   -2.26548600
13
Osmium Standard S0
 Os                -0.00332200    0.00370400   -0.06893500
 C                  0.09171600   -0.11049000    2.02973400
 C                 -1.45922800    1.47055400    0.24492600
 C                  1.48795100    1.45793300    0.11090900
 C                  1.46857000   -1.48072400   -0.04522700
 C                 -1.47900000   -1.46926500    0.08496800
 N                  0.14318600   -0.17664600    3.19536200
 N                  2.29650300   -2.30785300   -0.03127400
 N                 -2.30465600   -2.29421900    0.17194400
 N                  2.32460300    2.26989300    0.21348600
 N                 -2.27551400    2.29135700    0.41764300
 N                 -0.08448200    0.10115900   -1.85717300
 O                 -0.13813400    0.16557900   -3.01033600
13
Osmium TSSF S0
 Os                 0.04257400    0.07991800    0.01418600
 C                  0.63239500   -0.63116900    1.76113800
 C                 -1.60952900   -1.09450500    0.51968300
 C                 -1.21966300    1.67449100    0.42585300
 C                  1.54180500    1.48190900   -0.33280100
 C                  1.49006700   -1.31898900   -0.58910700
 N                  0.99557600   -0.98366600    2.82017000
 N                  2.36457400    2.30118800   -0.50065100
 N                  2.30913500   -2.07734800   -0.94505700
 N                 -1.90000800    2.60591700    0.64312800
 N                 -2.55237900   -1.72789800    0.80881800
 N                 -0.52907300   -0.56008100   -1.92607500
 O                 -1.63260600   -0.45637400   -2.26113400
13
Osmium Flat S0
 Os                -0.01899700    0.00069500   -0.09915700
 C                  0.32516900   -0.00983800    1.89604800
 C                 -0.15662800   -2.08461400   -0.07065900
 C                 -2.04190100    0.06650600    0.41250200
 C                 -0.01774800    2.09084700   -0.07143500
 C                  2.07594900   -0.06970600    0.02092700
 N                  0.48968700   -0.01454400    3.05369200
 N                 -0.03696200    3.26215300   -0.05113700
 N                  3.24249100   -0.10888200    0.09287700
 N                 -3.16930500    0.10296600    0.72290000
 N                 -0.25441000   -3.25199500   -0.05093100
 N                  0.52387400   -0.01645800   -2.02169600
 O                 -0.65411300    0.02191300   -2.22603500
13
Osmium TSFR S0
 Os                 0.12708000    0.02603800   -0.04245800
 C                  1.07919400    0.00762100    1.65806800
 C                 -1.36678800    1.09388800    0.89573100
 C                  0.97507600    1.82321600   -0.65020300
```

C	1.57714600	-1.00490400	-1.12313400
C	-0.58016200	-1.82925400	0.57950500
N	1.68790100	0.01289000	2.66500900
N	2.38825000	-1.57518200	-1.75467100
N	-0.97755300	-2.88051800	0.92214700
N	1.47657600	2.82959100	-0.99387200
N	-2.26344000	1.70526600	1.35051200
O	-2.06456200	-0.59810200	-1.21855300
N	-2.77579100	0.23117300	-1.50120600

13

Osmium Reverse S0

Os	0.00038000	-0.00225500	-0.02399500
C	0.04265900	0.11922000	1.99484400
C	-2.07834100	0.12120600	0.09758400
C	0.11764400	2.07974600	-0.05825000
C	2.08218300	-0.11643300	0.02633800
C	-0.11297000	-2.07428800	0.18786200
N	0.06849500	0.19069700	3.15347600
N	3.25195700	-0.15548000	0.06011400
N	-0.15890300	-3.22577200	0.31324400
N	0.16248000	3.25235300	-0.06693400
N	-3.25752000	0.17119000	0.17538200
O	-0.04173800	-0.11717800	-1.94183900
N	-0.06679300	-0.18554200	-3.08269900
